# Impact of a board certification system and implementation of clinical practice guidelines for pancreatic cancer on mortality of pancreaticoduodenectomy

**DOI:** 10.1007/s00595-020-02017-3

**Published:** 2020-05-07

**Authors:** Masamichi Mizuma, Hiroyuki Yamamoto, Hiroaki Miyata, Mitsukazu Gotoh, Michiaki Unno, Tooru Shimosegawa, Yasushi Toh, Yoshihiro Kakeji, Yasuyuki Seto

**Affiliations:** 1 Japan Pancreas Society, Osaka, Japan; 2 Department of Healthcare Quality Assessment, Graduate School of Medicine, The University of Tokyo, Tokyo, Japan; 3 The Japanese Society of Gastroenterological Surgery, Tokyo, Japan

**Keywords:** Pancreaticoduodenectomy, Pancreatic cancer, Quality indicator, Board certification, Clinical practice guideline

## Abstract

**Purposes:**

The aim of this study was to clarify the impact of a board certification system and the implementation of clinical practice guidelines for pancreatic cancer (PC) on the mortality of pancreaticoduodenectomy in Japan.

**Methods:**

By a web questionnaire survey via the National Clinical Database (NCD) for departments participating in the NCD, quality indicators (QIs) related to the treatment for PC, namely the board certification systems of various societies and the adherence to clinical practice guidelines for PC, were investigated between October 2014 and January 2015. A multivariable logistic regression analysis was performed to evaluate the relationship between the QIs and mortality of pancreaticoduodenectomy.

**Results:**

Of 1415 departments that registered at least 1 pancreaticoduodenectomy between 2013 and 2014 in NCD, 631 departments (44.6%), which performed pancreaticoduodenectomy for a total of 11,684 cases, answered the questionnaire. The mortality of pancreaticoduodenectomy was positively affected by the board certification systems of the Japanese Society of Gastroenterological Surgery, Japanese Society of Hepato-Biliary-Pancreatic Surgery, Japanese Society of Gastroenterology, and Japanese Society of Medical Oncology as well as by institutions that used magnetic resonance imaging of ≥ 3 T for the diagnosis of PC in principle.

**Conclusions:**

The measurement of the appropriate QIs is suggested to help improve the mortality in pancreaticoduodenectomy. Masamichi Mizuma and Hiroyuki Yamamoto equally contributed

## Introduction

Quality indicators (QIs) are utilized to measure the quality of care, which can be defined as “the degree to which health services for individuals and populations increase the likelihood of desired health outcomes and are consistent with current professional knowledge” [1]. The quality of care has been reported to be evaluated from three aspects: “structure”, “process” and “outcome” [2]. For example, board-certified experts are an indicator of structure. In addition, diagnostic methods or treatments recommended in a clinical practice guideline correspond to process indicators. The evaluation and improvement of the quality of care in each institution ultimately lead to the uniform accessibility of medical care nationwide. Therefore, assessing the quality of care using QIs is very important.

The registration of surgical cases in the National Clinical Database (NCD), which is linked to the board certification system of some surgical societies, began in 2011. Most surgical cases (90%-95%) performed in Japan are included in the NCD [3]. Approximately 10,000 cases of pancreaticoduodenectomy (PD), classified as having a high degree of difficulty in the surgical difficulty category defined by the Japanese Society of Gastroenterological Surgery (JSGS), are registered per year on NCD [4]. Risk models of the eight main procedures, including PD, were created using NCD data [5–12] and are used in the risk calculator on the NCD web site, which is available in clinical settings. PD is still a high-risk procedure, and the operative mortality and morbidity need improvement. The evaluation of QIs related to PD is thought to contribute to the improvement of the surgical outcome.

A questionnaire survey of the board certification system and the implementation of clinical practice guidelines for cancers of the esophagus, stomach, colorectum, liver, pancreas, biliary tract, lung and breast was conducted using the NCD to investigate their impact on the surgical mortality by a study group for “the utilization of high-accuracy organ cancer registration in the clinical practice guidelines and medical specialist training” and was supported by a grant from the Ministry of Health, Labour and Welfare of Japan. The results concerning esophageal and colon cancers have been recently reported [13, 14].

The present study aimed to elucidate the impact of the board certification system and the adherence to the clinical practice guideline for pancreatic cancer on mortality of PD.

## Methods

### Web questionnaire using the NCD registration system

**Fig. 1:**
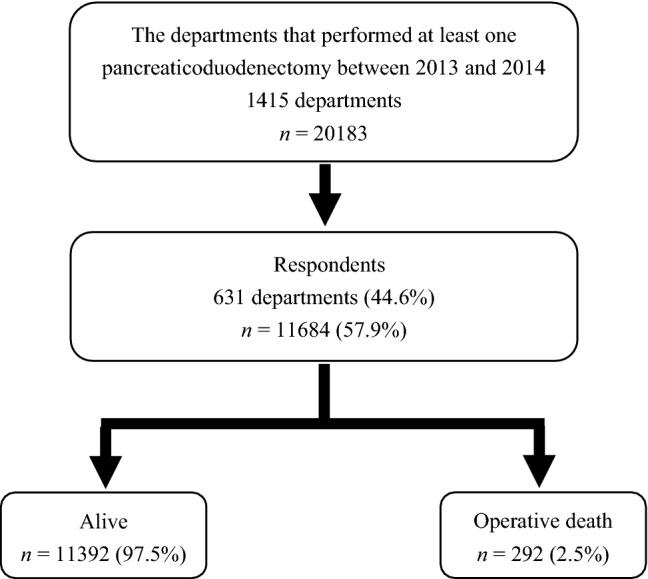
The flow chart of the patient selection process

The questionnaire form was created with the NCD registration system. The questionnaire survey of the QIs related to the treatment for pancreatic cancer was performed via the NCD web page between October 1, 2014, and January 31, 2015. The QIs of the questionnaire, which were chosen by discussion among experts on pancreatic diseases (MM, MU, TS and MG), are shown in Table 1. Q1–16, mainly asking whether or not there is a board-certified expert in each society related to the treatment of pancreatic cancer, were created as structure indicators. Q3 regarding board-certified institutions of the Japanese Society of Hepato-Biliary-Pancreatic Surgery (JSHBPS) was answered separately for Training Institutions A and B. In the application for the board certification system of JSHBPS, Training Institutions A and B need to perform 50 and 30 high-level hepato-biliary-pancreatic surgeries annually, as defined by the JSHBPS, respectively [15]. Board-certified experts of the Japanese Society of Gastroenterology (JSGE) or Japanese Society of Medical Oncology (JSMO), who are not necessarily surgeons, may participate in preoperative care for PD. Thus, the board certification systems of the JSGE and JSMO were considered for the questionnaire because they may affect the outcomes of PD. Q17–22 were selected as process indicators from Clinical Questions (CQs) of Clinical Practice Guidelines for Pancreatic Cancer Based on Evidence-Based Medicine 2013 [16]. The subjects of the questionnaire were a total of 1415 departments that performed at least 1 case of PD between 2013 and 2014, including a total of 20,183 PD cases in this study (Fig. 1).

**Table 1:** Questionnaire items related to the treatment of pancreatic cancer

Structure indicator	
Q1	Is your institution accredited by or related to the Japan Surgical Society (JSS)?
Q2	Is your institution certified by the Japanese Society of Gastroenterological Surgery (JSGS)?
Q3	Is your institution a board-certified training institution (Hepatobiliary-Pancreatic field) of the Japanese Society of Hepato-Biliary-Pancreatic Surgery (JSHBPS)?
Q4	Is your institution certified by or related to the Japanese Society of Gastroenterology (JSGE)?
Q5	Is your institution an accredited training facility of the Japanese Society of Medical Oncology (JSMO)?
Q6	Does your institution register cases of pancreatic cancer in the Japan Pancreatic Cancer Registry of the National Clinical Database (NCD)?
Q7	Does your institution have a board-certified instructor of JSS?
Q8	Does your institution have an expert surgeon of gastroenterological surgery board-certified by JSGS?
Q9	Does your institution have an instructor of gastroenterological surgery board-certified by JSGS?
Q10	Does your institution have a board-certified expert surgeon (Hepatobiliary-Pancreatic field) by JSHBPS?
Q11	Does your institution have an instructor (Hepatobiliary-Pancreatic field) board-certified by JSHBPS?
Q12	Does your institution have a gastroenterologist board-certified by JSGE?
Q13	Does your institution have an instructor of gastroenterology board-certified by JSGE?
Q14	Does your institution have an oncologist board-certified by JSMO?
Q15	Does your institution have an instructor of oncology board-certified by JSMO?
Q16	Does your institution have a General Clinical Oncologist certified by the Japanese Board of Cancer Therapy?
Process indicator	
Q17	Are contrast media used in CT or MRI to diagnose pancreatic cancer?
Q18	Is MRI of 3 T or more performed to diagnose pancreatic cancer?
Q19	Is radical resection performed for cases with Stage 0–IVa* pancreatic cancer without invasion of SMA or CA? Or does your institution refer them to other institutions for radical resection? *General Rules for the Study of Pancreatic Cancer the 6th Edition (the 3rd English Edition) by Japan Pancreas Society
Q20	Is S-1 monotherapy performed as the first choice in adjuvant chemotherapy for pancreatic cancer
Q21	Is chemoradiotherapy or chemotherapy performed as the first-line therapy for locally advanced unresectable pancreatic cancer?
Q22	Is either gemcitabine monotherapy, gemcitabine plus erlotinib combination therapy, or S-1 monotherapy performed as the first-line chemotherapy for locally advanced unresectable or metastatic pancreatic cancer?

*CA* celiac artery, *CT* computed tomography, *JSGE* Japanese Society of Gastroenterology, *JSGS* Japanese Society of Gastroenterological Surgery, *JSHBPS* Japanese Society of Hepato-Biliary-Pancreatic Surgery, *JSMO* Japanese Society of Medical Oncology, *JSS* Japan Surgical Society, *MRI* magnetic resonance imaging, *NCD* National Clinical Database, *SMA* superior mesenteric artery

### Operative mortality for each QI

Responses to the QI questionnaire were obtained from 631 departments (44.6%), which performed 11,684 pancreaticoduodenectomies (57.9%) during the study period. These PD cases were analyzed using the NCD database. Operative death was observed in 292 cases (2.5%) (Fig. 1). Operative death was defined as any death within the index hospitalization period up to 90 days after surgery or any death after discharge within 30 days after surgery. The operative mortality was analyzed for each QI of the questionnaire.

### The multivariable logistic regression analysis

The relationship between each QI of the questionnaires and operative death was analyzed by multivariable logistic regression models fitted with a generalized estimating equation, considering the clustering of patients by the hospital level. According to a previous report on the risk model using the NCD [5], the following variables were used to adjust risk factors by the patient background: age, respiratory distress (any), the activity of daily life (ADL) within 30 days before surgery (any assistance), angina, weight loss > 10%, American Society of Anesthesiologists (ASA) performance status grade ≥ Class 3, Brinkman index > 400, body mass index (BMI) > 25 kg/m^2^, serum creatinine > 3 mg/dl, platelet count < 120,000/μl, prothrombin time- international normalized ratio (PT-INR) > 1.1, white blood cell (WBC) count > 11,000/μl and activated partial thromboplastin time (APTT) > 40 s. Q1 and Q17 were excluded from the multivariable analysis because very few departments answered “no” and “Not performed in principle”, respectively.

### Statistical analyses

The STATA 15 software program (STATA Corp., College Station, TX, USA) was used for all statistical analyses. The significance of categorical variates was calculated using the chi-square test or Fisher’s exact test. The risk-adjusted odds ratio (AOR) and 95% confidence interval (CI) were calculated in multivariable logistic regression analyses. *P* < 0.05 was considered statistically significant.

This specific project was approved by the Ethics Committee of Fukushima Medical University (No. 1057).

## Results

### Patient demographics and crude operative mortality

The crude operative mortality was investigated for each risk factor according to the previous report of the risk model for PD (Table 2) [5]. All risk model variables except for a Brinkman index > 400 were significantly correlated with operative death.

**Table 2:** Preoperative factors and crude operative mortality rates

Variables	Operative death (*n* = 292)		Alive (*n* = 11,392)		*p* value
	*N*	%	*N*	%	
Age (years)					< 0.001
≤ 59	14	4.8	1726	15.2	
60–64	24	8.2	1558	13.7	
65–69	56	19.2	2132	18.7	
70–74	68	23.3	2541	22.3	
75–79	70	24.0	2134	18.7	
≥ 80	60	20.5	1301	11.4	
Respiratory distress (any)	5	1.7	82	0.7	0.067*
ADL within 30 days before surgery (any assistance)	25	8.6	263	2.3	< 0.001
Angina	12	4.1	118	1.0	< 0.001
Weight loss > 10%	30	10.3	634	5.6	0.001
ASA ≥ Class 3	87	29.8	1244	10.9	< 0.001
Brinkman index > 400	96	32.9	3511	30.8	0.452
BMI > 25 kg/m^2^	78	26.7	1789	15.7	< 0.001
Creatinine > 3 mg/dl	13	4.5	79	0.7	< 0.001
Platelet < 120,000/μl	19	6.5	352	3.1	0.001
PT-INR > 1.1	55	18.8	1308	11.5	< 0.001
WBC > 11,000/μl	13	4.5	260	2.3	0.015
APTT > 40 s	20	6.8	433	3.8	0.008

*ADL* activity of daily life, *APTT* activated partial thromboplastin time, *ASA* American Society of Anesthesiologists, *BMI* body mass index, *PT-INR* prothrombin time-international normalized ratio, *WBC* white blood cell

^*^Fisher's exact test

### The response distribution and crude operative mortality for each QI

Tables 3 and 4 indicate the response distribution and crude operative mortality rate in each QI for the structure and process indicators, respectively.

**Table 3:** The response distributions and relationship between each quality indicator and the crude operative mortality: structure indicator

Questionnaire item	Department (*N*)	Operative death (*n* = 292)		Alive (*n* = 11,392)		Total		Mortality rate
		*N*	%	*N*	%	*N*	%	
Q1 Institution accredited by or related to JSS								*p* = 0.162*
No	7	2	0.7	39	0.3	41	0.4	4.88%
Accredited	527	274	93.8	10,908	95.8	11,182	95.7	2.45%
Related	97	16	5.5	445	3.9	461	3.9	3.47%
Q2 Institution certified by JSGS								*p* < 0.001
Yes	493	256	87.7	10,626	93.3	10,882	93.1	2.35%
No	138	36	12.3	766	6.7	802	6.9	4.49%
Q3 A JSHBPS board-certified training institution								*p* < 0.001
No	473	163	55.8	4370	38.4	4533	38.8	3.60%
Training Institution A	96	81	27.7	5223	45.8	5304	45.4	1.53%
Training Institution B	62	48	16.4	1799	15.8	1847	15.8	2.60%
Q4 Institution certified by or related to JSGE								*p* < 0.001
No	112	35	12.0	716	6.3	751	6.4	4.66%
Accredited	425	230	78.8	10,010	87.9	10,240	87.6	2.25%
Related	94	27	9.2	666	5.8	693	5.9	3.90%
Q5 An accredited training facility of JSMO								*p* < 0.001
Yes	180	116	39.7	6201	54.4	6317	54.1	1.84%
No	451	176	60.3	5191	45.6	5367	45.9	3.28%
Q6 Registration in the Japan Pancreatic Cancer Registry of NCD								*p* = 0.574
All cases registered	449	210	71.9	8400	73.7	8610	73.7	2.44%
Some cases registered	113	45	15.4	1768	15.5	1813	15.5	2.48%
Not registered	69	37	12.7	1224	10.7	1261	10.8	2.93%
Q7 A JSS board-certified instructor								*p* = 1.000*
Yes	600	289	99.0	11,250	98.8	11,539	98.8	2.50%
No	31	3	1.0	142	1.2	145	1.2	2.07%
Q8 A JSGS board-certified expert surgeon of gastroenterological surgery								*p* = 0.408*
Yes	605	287	98.3	11,255	98.8	11,542	98.8	2.49%
No	26	5	1.7	137	1.2	142	1.2	3.52%
Q9 A JSGS board-certified instructor of gastroenterological surgery								*p* = 0.081
Yes	559	274	93.8	10,925	95.9	11,199	95.8	2.45%
No	72	18	6.2	467	4.1	485	4.2	3.71%
Q10 A JSHBPS board-certified expert surgeon								*p* < 0.001
Yes	120	89	30.5	4688	41.2	4777	40.9	1.86%
No	511	203	69.5	6704	58.8	6907	59.1	2.94%
Q11 A JSHBPS board-certified instructor								*p* < 0.001
Yes	241	164	56.2	7938	69.7	8102	69.3	2.02%
No	390	128	43.8	3454	30.3	3582	30.7	3.57%
Q12 A JSGE board-certified gastroenterologist								*p* = 0.004
Yes	560	270	92.5	10,923	95.9	11,193	95.8	2.41%
No	71	22	7.5	469	4.1	491	4.2	4.48%
Q13 A JSGE board-certified instructor of gastroenterology								*p* < 0.001
Yes	411	210	71.9	9310	81.7	9520	81.5	2.21%
No	220	82	28.1	2082	18.3	2164	18.5	3.79%
Q14 A JSMO board-certified oncologist								*p* = 0.001
Yes	179	113	38.7	5574	48.9	5687	48.7	1.99%
No	452	179	61.3	5818	51.1	5997	51.3	2.98%
Q15 A JSMO board-certified instructor of oncology								*p* = 0.004
Yes	191	120	41.1	5650	49.6	5770	49.4	2.08%
No	440	172	58.9	5742	50.4	5914	50.6	2.91%
Q16 A General Clinical Oncologist certified by the Japanese Board of Cancer Therapy								*p* = 0.232
Yes	563	276	94.5	10,928	95.9	11,204	95.9	2.46%
No	68	16	5.5	464	4.1	480	4.1	3.33%

*JSGE* Japanese Society of Gastroenterology, *JSGS* Japanese Society of Gastroenterological Surgery, *JSHBPS* Japanese Society of Hepato-Biliary-Pancreatic Surgery, *JSMO* Japanese Society of Medical Oncology, *JSS* Japan Surgical Society, *NCD* National Clinical Database

^*^Fisher's exact test

**Table 4:** The response distributions and relationship between each quality indicator and the crude operative mortality: process indicator

Questionnaire item	Department no.	Operative death (*n* = 292)		Alive (*n* = 11,392)		Total		Mortality rate
		*N*	%	*N*	%	*N*	%	
Q17 Contrast media in CT or MRI to diagnose pancreatic cancer								*p* = 0.258*
Performed in principle	592	281	96.2	10,931	96.0	11,212	96.0	2.51%
Not performed in principle	2	1	0.3	154	1.4	155	1.3	0.65%
Doctor's discretion	37	10	3.4	307	2.7	317	2.7	3.15%
Q18 MRI of 3 T or more to diagnose pancreatic cancer								*p* < 0.001
Performed in principle	285	119	40.8	6330	55.6	6449	55.2	1.85%
Not performed in principle	217	101	34.6	2843	25.0	2944	25.2	3.43%
Doctor's discretion	129	72	24.7	2219	19.5	2291	19.6	3.14%
Q19 Radical resection for cases with Stage 0–IVa* pancreatic cancer without invasion of SMA or CA, or referral to other institutions for radical resection * General Rules for the Study of Pancreatic Cancer the 6th Edition (the 3rd English Edition) by Japan Pancreas Society								*p* = 0.018
Performed in principle	463	238	81.5	9880	86.7	10,118	86.6	2.35%
Not performed in principle	34	14	4.8	490	4.3	504	4.3	2.78%
Doctor's discretion	134	40	13.7	1022	9.0	1062	9.1	3.77%
Q20 S-1 monotherapy as the first choice in adjuvant chemotherapy for pancreatic cancer								*p* = 0.001
Performed in principle	305	157	53.8	7205	63.2	7362	63.0	2.13%
Not performed in principle	95	36	12.3	1389	12.2	1425	12.2	2.53%
Doctor's discretion	231	99	33.9	2798	24.6	2897	24.8	3.42%
Q21 Chemoradiotherapy or chemotherapy as the first-line therapy for locally advanced unresectable pancreatic cancer								*p* = 0.549
Performed in principle	413	215	73.6	8701	76.4	8916	76.3	2.41%
Not performed in principle	35	15	5.1	535	4.7	550	4.7	2.73%
Doctor's discretion	183	62	21.2	2156	18.9	2218	19.0	2.80%
Q22 Either gemcitabine monotherapy, gemcitabine plus erlotinib combination therapy, or S-1 monotherapy as the first-line chemotherapy for locally advanced unresectable or metastatic pancreatic cancer								*p* = 0.291
Performed in principle	391	199	68.2	7644	67.1	7843	67.1	2.54%
Not performed in principle	31	10	3.4	630	5.5	640	5.5	1.56%
Doctor's discretion	209	83	28.4	3118	27.4	3201	27.4	2.59%

*CA* celiac artery, *CT* computed tomography, *MRI* magnetic resonance imaging, *SMA* superior mesenteric artery

^*^Fisher's exact test

As shown in Qs2–5, the board-certified institutions of the JSGS, JSHBPS, JSGE, and JSMO showed a significantly lower mortality rate than the non-certified institutions (*p* < 0.001). Regarding QIs related to board-certified experts or instructors, institutions having an expert surgeon and instructor board-certified by the JSHBPS (*p* < 0.001), a gastroenterologist and instructor board-certified by the JSGE (*p* = 0.004 and *p* < 0.001), and an oncologist and instructor board-certified by the JSMO (*p* = 0.001 and *p* = 0.004) showed significantly lower operative mortality than others (Table 3).

However, regarding QIs for process indicators, departments that used magnetic resonance imaging (MRI) of ≥ 3 T for the diagnosis of pancreatic cancer in principle (*p* < 0.001), performed radical resection for pancreatic cancer or referred the case to other institutions for radical resection in principle (*p* = 0.018) and performed S-1 adjuvant therapy for pancreatic cancer in principle (*p* = 0.001) showed a significantly lower operative mortality rate than others (Table 4). In Q18, 285 departments (45.2%) responded with “Performed in principle” concerning the use of MRI of ≥ 3 T for the diagnosis of pancreatic cancer.

### Results of the multivariable logistic regression analysis

**Fig. 2:**
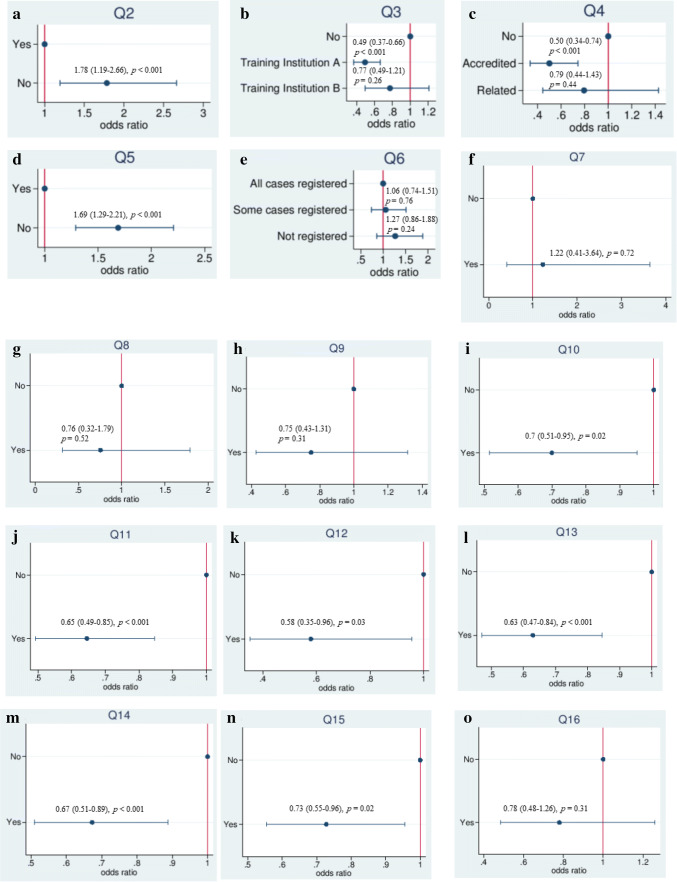
Relationship between each structure indicator and the AOR in PD. **a** Q2: Institution certified by the JSGS. **b** Q3: A training institution board-certified by the JSHBPS. **c** Q4: Institution certified by or related to the JSGE. **d** Q5: An accredited training facility of the JSMO. **e** Q6: Registration in the Japan Pancreatic Cancer Registry of the NCD. **f** Q7: A board-certified instructor of the JSS. **g** Q8: An expert surgeon of gastroenterological surgery board-certified by the JSGS. **h** Q9: An instructor of gastroenterological surgery board-certified by the JSGS. **i** Q10: An expert surgeon board-certified by the JSHBPS. **j** Q11: An instructor board-certified by the JSHBPS. **k** Q12: A gastroenterologist board-certified by the JSGE. **l** Q13: An instructor of gastroenterology board-certified by the JSGE. **m** Q14: An oncologist board-certified by the JSMO. **n** Q15: An instructor of oncology board-certified by the JSMO. **o** Q16: A General Clinical Oncologist certified by the Japanese Board of Cancer Therapy

**Fig. 3:**
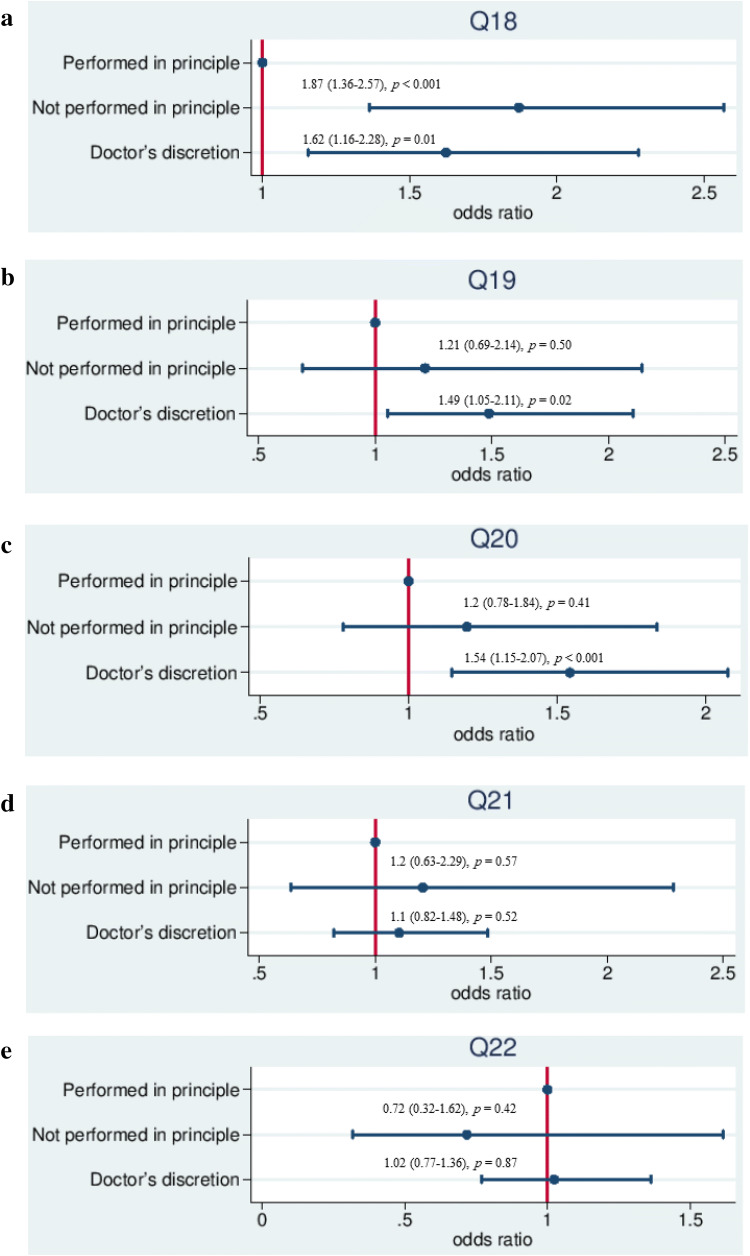
Relationship between each process indicator and the AOR in PD. **a** Q18: MRI of ≥ 3 T to diagnose pancreatic cancer. **b** Q19: Radical resection for cases with Stage 0–IVa* pancreatic cancer without invasion of SMA or CA, or referral to other institutions for radical resection. **c** Q20: S-1 monotherapy as the first choice in adjuvant chemotherapy for pancreatic cancer. **d** Q21: Chemoradiotherapy or chemotherapy as the first-line therapy for locally advanced unresectable pancreatic cancer. **e** Q22: Either gemcitabine monotherapy, gemcitabine plus erlotinib combination therapy, or S-1 monotherapy as the first-line chemotherapy for locally advanced unresectable or metastatic pancreatic cancer

Figure 2 and 3 show the AOR and 95% CI for each structure and process-related QI according to a multivariable logistic regression analysis with risk-adjustment using patient-level risk factors.

The AOR was significantly higher in institutions not certified by the JSGS (1.78 [1.19–2.66], *p* < 0.001) or JMSO (1.69 [1.29–2.21], *p* < 0.001) than in those that were certified (Fig. 2a, d). Compared with institutions that were not board-certified by the JSHBPS, JSHBPS board-certified training institution A showed a significantly lower AOR (0.49 [0.37–0.66], *p* < 0.001). In contrast, there was no significant difference between the JSHBPS board-certified training institution B and the institutions that were not board-certified (Fig. 2b). Although institutions accredited by the JSGE showed a significantly lower AOR (0.50 [0.34–0.74], *p* < 0.001) than those not certified by or related to the JSGE, related institutions showed no significant difference (Fig. 2c). Institutions with an expert surgeon or instructor board-certified by the JSHBPS (Fig. 2i, j), a gastroenterologist or instructor of gastroenterology board-certified by the JSGE (Fig. 2k, l), and an oncologist or instructor of oncology board-certified by the JSMO (Fig. 2m, n) showed significantly lower AOR values than those without them.

Regarding the use of MRI of ≥ 3 T for the diagnosis of pancreatic cancer (Q18), both “Not performed in principle” and “Doctor’s discretion” were significantly poor risk factors (*p* < 0.001 and *p* = 0.01) (Fig. 3a). Regarding radical resection (Q19) and S-1 adjuvant chemotherapy (Q20), “Doctor’s discretion” showed a significantly higher AOR than “Performed in principle”. “Not performed in principle” showed no significant difference in Q19 and Q20 (Fig. 3b, c).

## Discussion

The present study revealed the following three points using questionnaires and the data of the NCD: (1) Mortality of PD was positively affected by the institution certification systems of the JSGS, JSHBPS, JSGE and JSMO. (2) Institutions with an expert or instructor board-certified by the JSHBPS, JSGE or JSMO showed a low PD mortality. (3) The mortality of PD was low in institutions that used MRI of ≥ 3 T for the diagnosis of pancreatic cancer in principle. These findings suggest to be useful as a QI for PD in Japan.

According to the NCD, the operative mortality of PD between 2011 and 2012 was reported to be lowest in training institution A (board-certified by the JSHBPS) followed by institution B and non-certified institutions. In addition, the participation of an expert surgeon or instructor who was board-certified by the JSHBPS in PD resulted in a lower operative mortality compared to that with no such participation [17]. The current study, which analyzed NCD data collected between 2013 and 2014, also showed a similar impact of the board certification system of the JSHBPS on the operative mortality of PD. The board certification system of the JSHBPS is suggested to be a good QI in PD for pancreatic cancer.

In contrast, regarding the board certification system of the JSGS, the present study indicated no marked correlation between the operative mortality and the presence of a board-certified expert surgeon or instructor. A previous report showed that the number of expert surgeons board-certified by the JSGS was a surrogate marker of the operative mortality in eight main procedures, including PD [18]. The present study’s lack of an investigation of the number of expert surgeons board-certified by the JSGS might have been associated with the absence of a correlation with the operative mortality. In our study, a favorable outcome of PD was observed in institutions board-certified by the JSGE or JSMO. Furthermore, institutions with experts board-certified by the JSGE or JSMO who did not necessarily participate directly in PD still showed a significantly lower operative mortality for PD than in those without. These results suggest that institutions that specialize in gastroenterology or oncology have more favorable outcomes from surgery due to an indirect effect, as gastroenterologists and oncologists are involved in preoperative care, including oncological judgement, chemotherapy and nutritional management, for patients scheduled for PD. Therefore, these results imply that systematic multidisciplinary approach for preoperative care improves the safety of PD. There are no reports on the relationship between the operative mortality of PD and the board certification systems of the JSGE or JSMO. These are novel findings as factors related to the operative mortality of PD.

To our knowledge, there have been no reports concerning the implementation of clinical practice guidelines for pancreatic cancer, including associations with the mortality of PD. In the present study, institutions using MRI of ≥ 3 T in principle for the diagnosis of pancreatic cancer had a significantly lower mortality rate of PD than those who did not or did so only at the doctor’s discretion (Q18). Although adherence to Q18 was low compared with other QIs, this might be due to the considerable number of institutions unable to perform MRI of ≥ 3 T. Since possession of an MRI machine of ≥ 3 T depends on a hospital’s financial standing, the results of Q18 may reflect the effects of the hospital volume. Interestingly, the present study showed that QIs in radical resection (Q19) and S-1 adjuvant chemotherapy (Q20) had higher AORs for “Doctor’s discretion” than for “Performed in principle”. In a previous study in this project concerning esophageal cancer, similar results were found in some QIs [13]. These findings suggest the importance of organizational compliance with clinical practice guidelines for pancreatic cancer.

Despite patient selection bias due to old age, which may be considered a factor of non-operative indication, especially in elderly patients with comorbidity, this study demonstrated that age was a significant risk factor for mortality in PD, as previously reported [5]. Mortality following PD for elderly patients with pancreatic cancer has been reported to be affected by specific comorbidities (chronic obstructive pulmonary disease, chronic kidney disease, dementia and sepsis) as patient factors [19]. The present study was conducted with risk adjustment for various patient factors, including the age, as described in the Methods section. However, as a structure indicator, a previous report showed that non-teaching hospitals have a higher risk of PD mortality for elderly patients with pancreatic cancer than teaching hospitals [20]. The present study clarified the correlation between the mortality of PD and board certification systems of various academic societies as structure indicators. Thus, the assessment of structural indicators is crucial for reducing the mortality of PD.

The utilization of administrative claims data in Japan for the wide-scale measurement of QIs in the treatment of various cancers, namely colorectal, lung, stomach, liver, breast, prostate and cervical cancer, has been reported [21]. When comparing NCD data with administrative claims data, the advantage is that the impact of QIs on surgical outcomes can be analyzed, as in the current study project [13]. At clinical settings in Japan, the NCD Breast Cancer Registry is used to assess the QIs recommended by the clinical practice guidelines. Registered NCD users can compare the implementation rates of the QIs in their institutes with those of the national average on the NCD web site, which helps eliminate cancer care disparity. Thus, the NCD is a useful tool for evaluating QIs related to each type of cancer.

The limitations of this study are similar to those previously described [13]. First, we cannot exclude respondents’ bias in the questionnaire surveys. The respondents were users registered in the NCD and not necessarily representative of the department. In other words, the answers may not necessarily reflect the policies of the department. Second, we received no answer from more than half of the institutions. There may be differences in the implementation rate of QIs or the mortality of PD between respondents and non-respondents. Third, there may have been selection bias for the QIs, which were selected by discussion among experts of pancreatic diseases, as mentioned above. Finally, PD cases with diseases other than pancreatic cancer were included in this study.

In conclusion, the mortality of PD was positively impacted by the institutional certification systems of the JSGS, JSHBPS, JSGE and JSMO. Institutions with an expert or instructor who was board-certified by the JSHBPS, JSGE or JSMO showed a lower mortality rate of PD than those without such a staff member. Furthermore, institutions performing MRI of ≥ 3 T for the diagnosis of pancreatic cancer showed a lower mortality from PD than others. The NCD is a useful tool for evaluating the quality of cancer care, especially for analyzing the impact of QIs on surgical outcomes.

## Abbreviations

ADL: Activity of daily life
AOR: Adjusted odds ratio
APTT: Activated partial thromboplastin time
ASA: American Society of Anesthesiologists
BMI: Body mass index
CA: Celiac artery
CI: Confidence interval
CT: Computed tomography
JSGE: Japanese Society of Gastroenterology
JSGS: Japanese Society of Gastroenterological Surgery
JSHBPS: Japanese Society of Hepato-Biliary-Pancreatic Surgery
JSMO: Japanese Society of Medical Oncology
JSS: Japan Surgical Society
MRI: Magnetic resonance imaging
NCD: National Clinical Database
PD: Pancreaticoduodenectomy
PT-INR: Prothrombin time- international normalized ratio
QI: Quality indicator
SMA: Superior mesenteric artery
WBC: White blood cell
